# Poor sleep health is associated with older brain age: the role of systemic inflammation

**DOI:** 10.1016/j.ebiom.2025.105941

**Published:** 2025-09-30

**Authors:** Yuyang Miao, Jiao Wang, Xuerui Li, Jie Guo, Maria M. Ekblom, Shireen Sindi, Qiang Zhang, Abigail Dove

**Affiliations:** a Department of Geriatrics, Tianjin Medical University General Hospital, Tianjin, China; b Department of Medicine (Huddinge), Karolinska Institutet, Stockholm, Sweden; c Aging Research Center, Department of Neurobiology, Care Sciences and Society, Karolinska Institutet, Stockholm, Sweden; d National Clinical Research Center for Geriatrics, West China Hospital, Sichuan University, Chengdu, China; e Department of Nutrition and Health, China Agricultural University, Beijing, China; f Department of Physical Activity and Health, Swedish School of Sport and Health Sciences, Stockholm, Sweden; g Division of Physiotherapy, Department of Neurobiology, Care Sciences and Society, Karolinska Institutet, Stockholm, Sweden; h Division of Clinical Geriatrics, Department of Neurobiology, Care Sciences and Society, Karolinska Institutet, Stockholm, Sweden; i Ageing Epidemiology Research Unit (AGE), School of Public Health, Faculty of Medicine, Imperial College London, UK

**Keywords:** Sleep, Brain age, Magnetic resonance imaging, Inflammation, UK Biobank

## Abstract

**Background:**

Poor-quality sleep has been linked to increased dementia risk. We investigated the relationship between healthy sleep pattern and older brain age, and the extent to which this is mediated by systemic inflammation.

**Methods:**

The study included 27,500 adults from the UK Biobank (mean age 54.7 y, 54.0% female). The presence of five self-reported healthy sleep characteristics (early chronotype, 7–8 h daily sleep, no insomnia, no snoring, no excessive daytime sleepiness) were summed into a healthy sleep score (0–5 pts) and used to define three sleep patterns: healthy (≥4 pts), intermediate (2–3 pts), and poor (≤1 pt). Low-grade inflammation was estimated using the INFLA-score, a composite index of inflammatory biomarkers. After a mean follow-up of 8.9 y, brain age was estimated using a machine learning model based on 1079 brain MRI phenotypes and used to calculate brain age gap (BAG; i.e., brain age minus chronological age). Data were analysed using linear regression and generalised structural equation models.

**Findings:**

At baseline, 898 (3.3%) participants had poor sleep, 15,283 (55.6%) had intermediate sleep, and 11,319 (41.2%) had healthy sleep. Compared to healthy sleep, intermediate (β = 0.25 [0.11, 0.40], *P* = 0.010) and poor (β = 0.46 [0.05, 0.87], *P* < 0.001) sleep were associated with significantly higher BAG. In mediation analysis, INFLA-score mediated 6.81% and 10.42% of the associations between intermediate and poor sleep and higher BAG.

**Interpretation:**

Poor sleep health may accelerate brain ageing. This may be driven by higher levels of systemic inflammation.

**Funding:**

10.13039/501100008599Alzheimerfonden; Demensfonden; 10.13039/100010771Loo and Hans Osterman Foundation for Medical Research; the 10.13039/501100003170Knowledge Foundation; 10.13039/501100004359Swedish Research Council.

**Research in context**

**Evidence before this study**

Poor sleep has been associated with dementia, but it is unclear whether sleep disturbances contribute to the development of dementia or are rather a consequence of prodromal dementia. It is therefore relevant to consider the relationship between sleep and very early changes in brain ageing. We searched PubMed to identify cohort studies examining the relationship between sleep characteristics and markers of brain ageing. The search strategy included the terms “(sleep) AND (brain ageing OR brain aging OR brain age OR brain MRI).” Previous population-based studies have linked poor sleep characteristics to brain MRI differences such as brain atrophy, cortical thinning, reduced hippocampal volume, and poorer white matter microstructural integrity. However, there is currently very little evidence on the association between sleep and estimated brain age, and even less regarding the potential biological mechanisms underlying this relationship.

**Added value of this study**

In this large-scale prospective analysis of 27,500 individuals, we found that poor sleep health was related to older brain age, estimated using a machine learning model based on 1079 brain MRI phenotypes. Specifically, the gap between brain and chronological age grew by approximately 0.5 years for every 1-point decrease in healthy sleep score. These associations were more pronounced in males and participants who were below the age of 60 at baseline. Moreover, in mediation analysis, chronic inflammation (measured by a composite of C-reactive protein, white blood cell count, platelet count, and granulocyte-to-lymphocyte ratio) mediated >10% of the association between poor sleep and older brain age. Taken together, these findings provide strong evidence that poor sleep may contribute to accelerated brain ageing and point to inflammation as one of the likely underlying biological mechanisms.

**Implications of all the available evidence**

Having an older brain age is an early indicator of a departure from optimal brain health. Our findings relating poor sleep to older brain age support the notion that poor sleep may be a risk factor for the development of dementia. Future studies are necessary to determine whether improving sleep characteristics can prolong brain and cognitive health.

## Introduction

Sleep is a fundamental behaviour that plays an essential role in a wide range of biological functions including regulation of metabolism, modulation of immune function, brain waste clearance, and memory consolidation.^1^ Disturbances in sleep are common in older age,^2^ and increasing evidence points to a complex relationship between sleep and dementia. On one hand, the neurodegeneration that characterises Alzheimer's disease and other dementias can lead to dysregulation of the sleep–wake cycle.^3^ Conversely, sleep disturbances may also contribute to the pathogenesis of dementia,^3^ with several population-based studies linking unhealthy sleep characteristics among cognitively healthy older adults—including excessively short or long sleep duration, insomnia, later rising, and daytime sleepiness^4^, ^5^, ^6^, ^7^, ^8^, ^9^, ^10^—to subsequent cognitive decline and incident dementia. To better understand poor sleep health as a potential risk factor for dementia, it is relevant to consider the relationship between sleep health and very early changes in brain health, before the symptoms of dementia set in.

In recent years, modelling methods have been introduced to estimate brain age based on brain magnetic resonance imaging (MRI) features such as volume loss, cortical thinning, white matter degradation, loss of gyrification, and ventricle enlargement.^11^ Brain age gap (BAG) refers to the discrepancy between an individual's brain age and their chronological age.^11^ Having an older-appearing brain relative to one's chronological age—i.e., high BAG—can be a sign of departure from the normal ageing process and has been associated with increased mortality and significantly elevated risk of cognitive decline and dementia, as well as other neurological disorders.^11^

Several studies have associated unhealthy sleep with specific components of brain age such as brain atrophy, cortical thinning, reduced hippocampal volume, and poorer white matter microstructural integrity,^12^, ^13^, ^14^, ^15^, ^16^ and a growing literature has linked prolonged sleep deprivation and disorders like sleep apnoea and sleep-disordered breathing to accelerated brain ageing.^17^, ^18^, ^19^ However, the relationship between overall sleep health and brain age has so far been addressed in only one small study.^20^

Given the heterogeneity of the ageing population, another important consideration is how clinically relevant factors such as age, sex, and genetic predisposition for dementia might modify the association between sleep health and brain age. Further research is also needed to understand the biological mechanisms connecting sleep and brain health. One candidate mechanism of particular interest is inflammation, given increasing evidence that sleep disturbances promote systemic inflammation and the role of inflammation as a driver of a range of neuropathologies including cerebrovascular disease, brain amyloid accumulation, and neurodegeneration.^21^, ^22^, ^23^

The present study comprehensively investigates the relationship between sleep health and brain ageing, leveraging detailed neuroimaging data from >27,000 middle-aged and older adults from the UK Biobank. Specifically, we aimed to 1) examine the association between sleep health and BAG; 2) explore the role of age, sex, and *APOE* ɛ4 in these associations; and 3) investigate the potential mediating role of inflammation in the sleep-BAG association. We hypothesise that poor sleep health is associated with faster brain ageing, and that inflammation may play a role in this association.

## Methods

### Study design and population

The UK Biobank is an ongoing longitudinal study including >500,000 adults aged 40–70 y from across the United Kingdom.^24^ The baseline examination took place between 2006 and 2010 at one of 22 assessment centres across the country and consisted of clinical assessments, a series of sociodemographic and lifestyle questionnaires, and collection of blood samples. Approximately 9 years later, between 2014 and 2020, 42,806 participants additionally underwent a brain MRI scan.

The present study used a sample of 27,500 participants for all analyses. Selection of the study sample is illustrated in Figure S1. Briefly, the analysis was first restricted to 34,296 participants who underwent brain MRI scans and had complete information available on all imaging phenotypes. We then excluded 1459 participants with chronic neurological disorders (including dementia, stroke, etc.; Table S1) at the time of MRI scan and 5582 with missing information on sleep.

### Ethics

All data used in this study were obtained from the UK Biobank through application #67048. Data collection procedures were approved by the UK National Research Ethics Service (Ref 11/NW/0382) and use of the data for the present analyses was additionally approved by the Regional Ethical Review Board in Stockholm (2024-00520-01).

### Assessment of sleep patterns

To comprehensively capture overall sleep patterns, rather than individual sleep characteristics, sleep was operationalised using a composite indicator^25^^,^^26^ based on the following parameters self-reported during the baseline examination: chronotype, sleep duration, insomnia, snoring, and daytime sleepiness. Participants were instructed to answer the questions according to how they have slept over the past month.

#### Chronotype

Participants self-reported their chronotype preference as one of the following: (1) definitely a “morning” person; (2) more a “morning” than “evening” person; (3) more an “evening” than “morning” person; or (4) definitely an “evening” person.

#### Sleep duration

Participants reported the number of hours of sleep they get in a typical day (including naps). Responses were recorded as whole numbers.

#### Insomnia

Based on the question “Do you have trouble falling asleep at night or do you wake up in the middle of the night?”, insomnia was classified as never/rarely, sometimes, or usually.

#### Snoring

Snoring behaviour (yes or no) was dichotomised based on the question “Does your partner or a close relative or friend complain about your snoring?”

#### Subjective daytime sleepiness

Based on the question “How likely are you to doze off or fall asleep during the daytime when you don't mean to? (e.g. when working, reading, or driving),” subjective daytime sleepiness was classified as never/rarely, sometimes, often, or all the time.

Following previous literature,^25^ a healthy sleep score was calculated based on the number (0–5) of healthy sleep factors present: early chronotype (‘morning’ or ‘more morning than evening’), 7–8 h sleep duration; no insomnia symptoms (‘never/rarely’); no snoring; and infrequent daytime sleepiness (‘never/rarely’ or ‘sometimes’). A categorical indicator of sleep health was then generated as follows: healthy sleep pattern (score ≥4), intermediate sleep pattern (score 2–3), poor sleep pattern (score ≤1).^25^

### Estimation of brain age and brain age gap

A mean of 8.9 years after the baseline assessment, brain MRI scans were conducted using Siemens Skyra 3T scanners at one of four imaging centres located in Manchester, Reading, Cheadle, and Newcastle. The UK Biobank brain MRI image acquisition and processing protocols are summarised in Table S2.^27^^,^^28^ A total of 1079 imaging-derived phenotypes (IDPs) were extracted across a total of six brain MRI modalities: 165 from T1-weighted MRI, 1 from T2-FLAIR, 14 from T2∗, 675 from diffusion MRI, 210 from resting-state fMRI, and 14 from task fMRI.

The procedure for brain age estimation has been described in our previous studies^29^^,^^30^ and is summarised in detail in Appendix I. Briefly, a subset of healthy participants was used to train and validate candidate machine learning models for estimating brain age, based on Z-scores for all IDPs. Among nine tested approaches, a LASSO regression model without feature selection performed best (Table S3) and was therefore used to predict brain age for the entire sample of participants with complete data available on all IDPs. In this model, 285 IDPs contributed significantly to the brain age estimate (Table S4).

BAG represents the difference between an individual's brain age and their chronological age and was calculated as follows: *BAG* = *brain age*—*age*_*time of MRI*_. Positive values for BAG indicate a brain that is older (i.e., less healthy) and negative values for BAG indicate a brain that is younger (i.e., more healthy) than expected based on the individual's chronological age.

### Calculation of INFLA-score

Low-grade inflammation was assessed using the INFLA-score, a composite index based on four inflammatory biomarkers collected from the baseline blood sample: C-reactive protein (CRP), white blood cell count, platelet count, and granulocyte-to-lymphocyte ratio (GrL).^31^, ^32^, ^33^ Each biomarker was divided into deciles and scored from −4 (1st decile) to +4 (10th decile), with intermediate deciles scored accordingly. The sum of these scores yielded a total INFLA-score ranging from −16 to +16, with higher values indicating greater inflammation.

### Assessment of covariates

Information on the following covariates was collected during the baseline examination:

#### Sociodemographic factors

Biological sex was self-reported. Education level was self-reported and dichotomised according to whether participants had completed college/university. Socioeconomic status (SES) was assessed using the Townsend Deprivation Index (TDI), a measure of neighbourhood-level socioeconomic deprivation based on the prevalence of unemployment, household overcrowding, and car/home ownership in a given postcode of residence.^34^ Race and ethnicity were self-reported according to the 2001 UK census categories and dichotomised as white vs. non-white (including Asian, Black, multiracial, or other).

#### Healthy lifestyle

Participants were classified as having a healthy lifestyle if they had three or more of the following: nonsmoking, no or light/moderate drinking, high physical activity, and/or high social contact.

Smoking status was categorised as nonsmoking, former smoker, or current smoker according to self-report. Alcohol consumption (in UK alcohol units; 1 unit = 8 g ethanol^35^) was categorised as no drinking, light/moderate drinking (≤14 units/week), or heavy drinking (>14 units/week) according to current UK guidelines.^35^ Regular physical activity was defined as at least 150 min of moderate–intensity activity per week, 75 min of vigorous activity per week, or a combination of equivalent activities.^36^ Social contact was estimated based on responses to the question “How often do you visit friends or family or have them visit you?” and categorised as high (almost daily, 2–4 times a week, about once a week) or low (about once a month, once every few months, or never/almost never).

#### Cardiometabolic disease

Cardiometabolic disease was defined as the presence of coronary artery disease, atrial fibrillation, heart failure, hypertension, and/or type 2 diabetes. Diagnoses were ascertained from medical records, medication use, and self-reported medical history.

#### APOE ɛ4

*APOE* was genotyped from blood samples collected at baseline and categorised as carriers vs. non-carriers of the ɛ4 allele.

### Statistical analysis

Baseline characteristics of the study participants by sleep pattern were assessed using χ^2^ tests for categorical variables and one-way analysis of variance (ANOVA) for continuous variables.

Linear regression models were used to estimate β-coefficients for the associations of healthy sleep score (continuous) and sleep pattern (categorical) with BAG. Additional models were conducted using the five individual sleep characteristics included in the healthy sleep score as the exposure. Least squares (LS) means of BAG (in years) were also estimated from the margins of each model.

Next, stratified linear regression models were used to explore the role of age (<60 y vs. ≥60 y), sex (male vs. female), and *APOE* ɛ4 status (carrier vs. non-carrier) in the association between sleep pattern and BAG. Interactions between sleep pattern and age, sex, and APOE ɛ4 status were assessed by incorporating the cross-product term into the models.

The relationship between INFLA-score and BAG was modelled using restricted cubic splines with four knots at fixed intervals of the INFLA-score distribution (−4, 0, 4, and 8). Finally, generalised structural equation models (GSEM) were used to analyse the mediating role of INFLA-score in the association between sleep pattern and BAG. Direct, indirect, and total effects were estimated with confidence intervals through bias-corrected bootstrapping (500 replications).

All models were first basic-adjusted for age, sex, and education level, followed by further adjustment for race, SES, healthy lifestyle, and cardiometabolic disease.

In sensitivity analysis, we repeated the main analyses after 1) using inverse probability weighting to account for the relatively lower prevalence of participants with poor sleep health in the study sample, 2) imputing missing values for covariates using multiple imputation by chained equations, 3) excluding 470 (1.7%) participants with baseline sleep disorders (ICD-10 codes F51 and G47; including sleep apnoea, restless leg syndrome, etc.), 4) excluding 2877 (10.5%) participants who were included in the training set for the brain age estimation model, and after additionally adjusting for 5) brain MRI assessment centre, 6) BMI, and 7) *APOE* ɛ4.

All analyses were performed using Python version 3.8.0 and Stata SE 16.0. All *P*-values presented were estimated from Wald tests; values < 0.05 were considered statistically significant.

### Role of funders

The funders had no role in the design, data collection, data analysis/interpretation, or writing of this study.

## Results

### Baseline characteristics of the study sample

Baseline characteristics of the 27,500 study participants (mean age 54.7 ± 7.5; 54.0% female) are summarised in Table 1. At baseline, 898 (3.3%) participants had a poor sleep pattern, 15,283 (55.6%) had an intermediate sleep pattern, and 11,319 (41.2%) had a healthy sleep pattern. Compared to participants with a healthy sleep pattern, those with an intermediate or poor sleep pattern were more likely to be older, male, and have lower SES, higher BMI, and prevalent cardiometabolic diseases. They were also less likely to have a college degree and a healthy lifestyle. Similar results were observed when participants were instead grouped by healthy sleep score (Table S5).

**Table 1:** Baseline characteristics of the study sample by sleep pattern.

Characteristics	Whole sample (n = 27,500)	By sleep pattern			
		Poor (n = 898)	Intermediate (n = 15,283)	Healthy (n = 11,319)	*P*-value
Age at baseline (years)	54.69 ± 7.49	54.6 ± 7.18	55.00 ± 7.35	54.27 ± 7.67	<0.001
Age at MRI assessment (years)	63.58 ± 7.63	63.4 ± 7.38	63.88 ± 7.50	63.19 ± 7.81	<0.001
Sex					<0.001
Female	14,861 (54.04)	406 (45.20)	7995 (52.31)	6460 (57.07)	
Male	12,639 (45.96)	492 (54.80)	7288 (47.69)	4859 (42.93)	
White	25,454 (92.74)	828 (92.30)	14,126 (92.62)	10,500 (92.94)	0.547
Townsend deprivation index	−2.65 (−3.9, −0.59)	−2.41 (−3.71, 0.02)	−2.62 (−3.89, −0.50)	−2.71 (−3.93, −0.75)	<0.001
Education (college)	12,829 (46.75)	345 (38.50)	6829 (44.78)	5655 (50.07)	<0.001
BMI (kg/m^2^)	26.46 ± 4.16	28.37 ± 4.98	26.86 ± 4.23	25.78 ± 3.86	<0.001
Healthy lifestyle	4800 (20.38)	121 (16.37)	2538 (19.35)	2141 (22.09)	<0.001
Non-smoking	16,648 (60.65)	456 (50.78)	8882 (58.24)	7310 (64.67)	<0.001
No heavy drinking	1521 (6.27)	60 (7.73)	804 (5.93)	657 (6.61)	0.023
Regular physical activity	19,936 (74.68)	560 (65.34)	10,804 (73.01)	8572 (77.63)	<0.001
High level of social contact	10,735 (39.1)	352 (39.29)	5939 (38.94)	4444 (39.29)	0.842
Cardiometabolic disease	12,517 (45.52)	479 (53.34)	7358 (48.14)	4680 (41.35)	<0.001
Coronary artery disease	728 (2.65)	37 (4.12)	459 (3.00)	232 (2.05)	<0.001
Atrial fibrillation	273 (0.99)	11 (1.22)	169 (1.11)	93 (0.82)	0.054
Heart failure	36 (0.13)	0 (0.00)	25 (0.16)	11 (0.10)	0.182
Hypertension	12,092 (43.97)	462 (51.45)	7116 (46.56)	4514 (39.88)	<0.001
Type 2 diabetes	705 (2.56)	43 (4.79)	428 (2.80)	234 (2.07)	<0.001
*APOE* ɛ4 carrier	6400 (27.6)	210 (27.45)	3555 (27.63)	2635 (27.57)	0.991
INFLA-score	−1.00 (−5.00, 3.00)	0.00 (−5.00, 4.00)	−1.00 (−5.00, 3.00)	−2.00 (−6.00, 2.00)	<0.001

Data are presented as means ± standard deviations or number (proportion, %). *P*-values were estimated from Wald tests.

Abbreviations: BMI, body mass index; *APOE*, *apolipoprotein* E.

Missing data: Education = 61 (0.2%); Race = 54 (0.2%); Townsend deprivation index = 23 (0.1%); Smoking = 49 (0.2%); Alcohol consumption = 5 (0.02%); Regular physical activity = 804 (2.9%); Regular social contact = 44 (0.2%); *APOE* ɛ4 = 4311 (15.7%); Inflammation score = 3040 (11.1%).

### Sleep pattern and brain age gap

As a continuous variable, lower healthy sleep score was associated with significantly higher BAG (β = 0.13 [0.06, 0.20], *P* < 0.001; Table 2), such that the gap between brain age and chronological age grew 0.48 years with each 1-point decrease in healthy sleep score. Consistent with this, BAG was significantly higher among participants with an intermediate (β = 0.24 [0.11, 0.38], *P* = 0.010) or poor (β = 0.50 [0.12, 0.87], *P* < 0.001) sleep pattern compared to those with a healthy sleep pattern. Specifically, brain age was on average 0.62 years older than chronological age among people with an intermediate sleep pattern and 0.99 years older among those with a poor sleep pattern.

**Table 2:** Standardised β coefficient and 95% confidence interval (CI) for the association between healthy sleep pattern and brain age gap (BAG): results from linear regressions.

	No. of participants	LS Mean ± SE	BAG			
			Basic adjusted		Multi-adjusted	
			β (95% CI)	*P*-value	β (95% CI)	*P*-value
Healthy sleep score (per 1-point decrease)	27,500	0.48 ± 0.03	0.19 (0.12, 0.25)	<0.001	0.13 (0.06, 0.20)	<0.001
5	2313	0.21 ± 0.11	Reference	–	Reference	–
4	9006	0.27 ± 0.08	0.06 (−0.17, 0.29)	0.602	−0.01 (−0.26, 0.24)	0.930
3	10,413	0.57 ± 0.05	0.31 (0.08, 0.53)	0.008	0.22 (−0.03, 0.46)	0.087
2	4870	0.73 ± 0.08	0.47 (0.22, 0.72)	<0.001	0.28 (0.01, 0.55)	0.045
0–1	898	0.99 ± 0.19	0.69 (0.30, 1.07)	0.001	0.49 (0.06, 0.91)	0.025
Sleep pattern						
Healthy	11,319	0.26 ± 0.05	Reference	–	Reference	–
Intermediate	15,283	0.62 ± 0.04	0.31 (0.19, 0.42)	<0.001	0.24 (0.11, 0.38)	0.010
Poor	898	0.99 ± 0.19	0.64 (0.29, 0.98)	<0.001	0.50 (0.12, 0.87)	<0.001

Basic-adjusted models included age, sex, and education. Multi-adjusted models additionally included race, socioeconomic status, healthy lifestyle (smoking status, alcohol intake, physical activity, and social contact), and cardiometabolic disease. LS (least squares) Mean and SE (standard error) were estimated from Model 2. *P*-values were estimated from Wald tests.

When we considered individual components of the healthy sleep score, late chronotype (β = 0.23 [0.09, 0.36], *P* = 0.001), abnormal sleep duration (β = 0.26 [0.12, 0.41], *P* < 0.001), and snoring (β = 0.14 [0.01, 0.28], *P* = 0.042) were most strongly associated with higher BAG (Table S6).

### Analyses stratified by age, sex, and APOE ɛ4 status

**Fig. 1:**
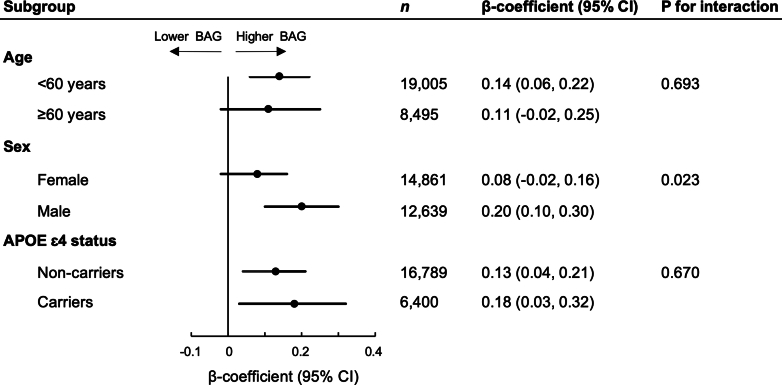
Subgroup analysis for the associations between healthy sleep score and brain age gap (BAG). Models were adjusted for age, sex, education, race, socioeconomic status, healthy lifestyle (smoking status, alcohol intake, physical activity, and social contact), and cardiometabolic disease. *P*-values were estimated from Wald tests. See Table S5 for the associations between sleep pattern (healthy, intermediate, poor) and BAG in each subgroup.

Subgroup analyses stratified by age, sex, and *APOE* ɛ4 status are presented in Fig. 1 (Table S7). The association between lower healthy sleep score and higher BAG was more pronounced among males (β = 0.20 [0.10, 0.30], *P* < 0.001) compared to females (β = 0.08 [−0.02, 0.16], *P* = 0.150) (P for interaction = 0.023). In contrast, the magnitude of the association between lower healthy sleep score and higher BAG was consistent across participants <60 y and ≥60 y (*P* for interaction = 0.693) and across *APOE* ɛ4 carriers and non-carriers (*P* for interaction = 0.670).

### The mediating role of inflammation

**Fig. 2:**
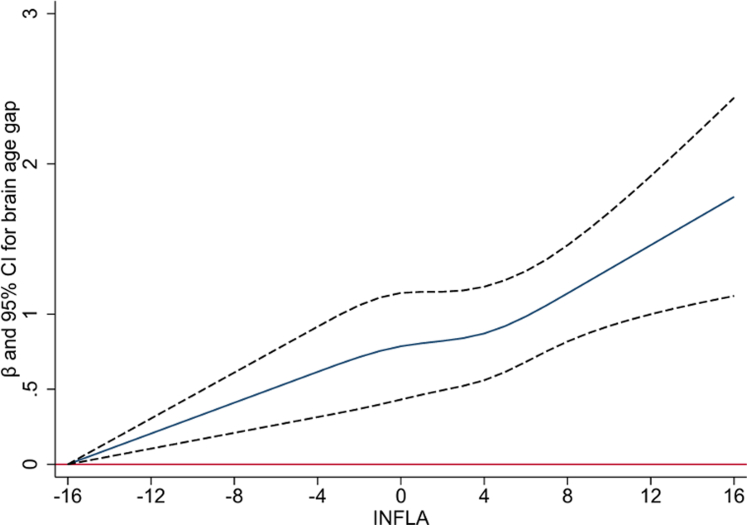
Association between chronic low-grade inflammation (INFLA-score) and brain age gap. The association between INFLA-score and brain age gap was analysed using linear regression with restricted cubic splines with 4 knots (−4, 0, 4, and 8). The model was adjusted for age, sex, education, race, socioeconomic status, healthy lifestyle (smoking status, alcohol intake, physical activity, and social contact), and cardiometabolic disease.

**Fig. 3:**
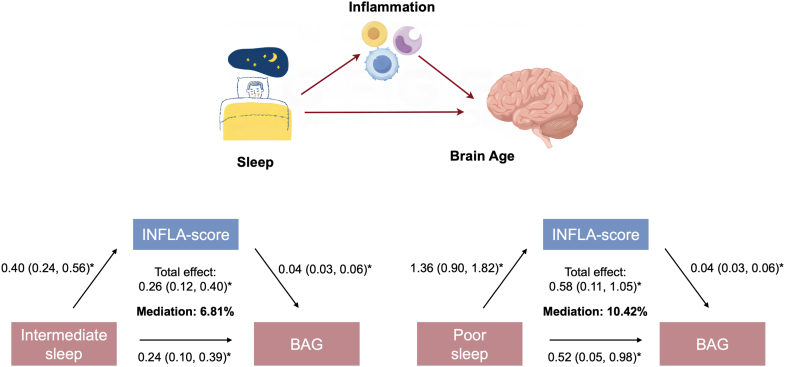
Mediation effect of chronic low-grade inflammation (INFLA-score) on the associations between sleep pattern and brain age gap (BAG). Models were adjusted for age, sex, education, race, socioeconomic status, healthy lifestyle (smoking status, alcohol intake, physical activity, and social contact), and cardiometabolic disease. Bootstrapping with 500 iterations was used to estimate the confidence intervals of mediation effects.

Restricted cubic splines showed a steep increase in BAG with higher INFLA-scores (Fig. 2; Figure S2). In mediation analysis, INFLA score mediated 6.81% of the association between intermediate sleep and higher BAG (*P* = 0.020) and 10.42% of the association between poor sleep and higher BAG (*P* = 0.009) (Fig. 3).

### Sensitivity analyses

In sensitivity analysis, similar results were obtained when we repeated the analyses using inverse probability weighting (Table S8), after imputing missing values for covariates (Table S9), after excluding individuals with sleep disorders (Table S10) and those who were included in the original brain age estimation model training set (Table S11), and after additionally adjusting for brain MRI assessment centre (Table S12), BMI (Table S13), and APOE ɛ4 (Table S14).

## Discussion

In this large-scale neuroimaging study, having an intermediate or poor sleep pattern was related to older brain age compared to chronological age. These associations were more pronounced in males and appear to be partially mediated by systemic inflammation.

Using a multimodal MRI-based measure of brain age, we found that BAG grew by approximately 0.5 years for every 1-point decrease in healthy sleep score, such that brain age was on average 0.6 years older than chronological age among people with an intermediate sleep pattern and 1 year older among people with a poor sleep pattern. Having a brain age that exceeds chronological age is an early indicator of a departure from optimal brain health. The fact that we observed meaningful differences in BAG as a function of sleep in this cognitively healthy population supports the notion that poor sleep may contribute to the development of dementia—a finding that has been suggested in several population-based studies.^4^, ^5^, ^6^, ^7^, ^8^, ^9^, ^10^ Of course, fully understanding the temporal relationship between sleep health and brain health will require further studies that integrate longitudinal measures of sleep, brain pathology, and cognitive status.

Our results are in line with a recent, but much smaller (n = 589), investigation from the CARDIA study, in which participants with poor sleep characteristics in early midlife had a late midlife brain age that was on average 2.6 years older than their counterparts with healthy sleep characteristics.^20^ Our findings are also consistent with previous studies on the relationship between individual sleep characteristics and structural brain MRI measures. In population-based studies such as the Framingham Heart Study, the Baltimore Longitudinal Study of Ageing, and the UK Biobank, having an excessively short or long sleep duration was related to cortical thinning,^13^ brain atrophy,^12^^,^^15^^,^^16^ reduced hippocampal volume,^15^^,^^16^ and poorer white matter microstructural integrity.^16^ In another report from the Irish Longitudinal Study on Aging, both abnormal sleep duration and sleep problems (i.e., daytime slippiness, trouble falling asleep, and interrupted sleep) were associated with smaller volumes in several hippocampal subfields.^14^

Our study goes a step beyond this literature by using a comprehensive measure of sleep health that reflects the combined role of five inter-related characteristics: chronotype, sleep duration, insomnia, snoring, and subjective daytime sleepiness.^25^ Sleep characteristics are highly inter-related with one another and can interact synergistically—for example, frequent insomnia could lead to excessive daytime sleepiness, and late chronotype could lead to reduced sleep duration. Therefore, by using a composite measure reflecting multiple aspects of sleep, our study more fully captures the complexity of sleep health—an important consideration in the older population, where sleep disturbances are especially prevalent.^2^

Considering the heterogeneity of the older population, we additionally investigated the role of a variety of other clinically relevant factors in the relationship between sleep and brain age. In stratified analyses, the association between lower healthy sleep score and higher BAG was more pronounced in males compared to females, but remained consistent between carriers and non-carriers of the APOE ɛ4 allele and between participants <60 y and ≥60 y (though the association became non-significant in the ≥60 y group, perhaps due to the greater clinical heterogeneity in this strata). The stronger association between sleep and brain health in males is rather surprising in light of the elevated dementia risk^37^ and higher prevalence of self-reported poor sleep quality^38^ among females. Possible sex differences in the relationship between sleep and brain health warrant deeper examination in future studies. Relevant areas for future exploration include whether the self-reported sleep measure equally captures sleep disturbances in men and women, the role of the menopause transition and related sleep problems during this period, and possible differences in drug use between men and women that may modify the sleep-BAG association.

To explore the potential mechanisms underlying the association between sleep and brain age, we conducted a mediation analysis, finding that systemic inflammation (as measured by INFLA-score) accounts for over 10% of the association between poor sleep and higher BAG. This is consistent with a large literature linking unhealthy sleep characteristics—including insomnia, sleep fragmentation, insufficient sleep, and sleep-disordered breathing—to inflammation, and in turn linking systemic inflammation to neuropathologies ranging from cerebrovascular disease to brain amyloid accumulation to neurodegeneration.^22^^,^^23^ Other complementary explanations for the connection between sleep and brain health centre on the glymphatic system (the brain's waste clearance system, which is active mainly during sleep) and the role of poor sleep in worsening existing risk factors for brain ageing, like cardiovascular disease.^23^ Mapping these potentially overlapping mechanisms in more detail is a rich area for future investigation.

Strengths of this study include the large sample, the use of a comprehensive machine-learning-based measure of brain age based on multimodal brain MRI data, and the inclusion of several inflammatory biomarkers in a mediation analysis to explore potential biological mechanisms for the observed association between sleep and BAG. However, some limitations should be acknowledged. First, the UK Biobank study population is highly selected and is composed of people who are substantially healthier and more socioeconomically advantaged than the general UK population.^39^^,^^40^ This could limit the generalisability of our findings and likely contributed to an underestimation of the true associations between sleep and brain age. Selection bias may be especially strong within the UK Biobank neuroimaging cohort^41^ due to the 9-year gap between baseline and the MRI scan (i.e., survival bias) and the exclusion of less healthy individuals from the MRI protocol due to contraindications like stents or pacemakers. Another limitation is that information on sleep was self-reported, introducing the possibility of information bias. Specifically, participants who live alone may have provided less accurate responses about snoring behaviour, and the sleep questionnaire may not have adequately captured “social jetlag”—i.e., potential mismatch between the circadian and social clock that can lead to differences in sleep duration and quality on weekdays vs. weekends and working vs. non-working days.^42^ Along similar lines, though participants were instructed to answer the sleep questionnaire based on their sleep over the past month, it is possible that they focused more on sleep during the previous night. This may have led to less temporal separation between sleep and the collection of inflammatory biomarkers, a key assumption of the mediation analysis. Previous reviews have suggested that there may be considerable discrepancies between self-reported sleep quality and data collected through polysomnography,^43^ so future studies integrating objective measures of sleep health are warranted.

In conclusion, the present study provides evidence that adverse sleep health may contribute to accelerated brain aging. The associations between poor sleep and higher BAG were more pronounced in males, and up to 10% of the sleep-BAG association may be mediated by systemic inflammation. Taken together, our findings highlight sleep as a possible modifiable factor that may influence brain health.

## Contributors

YM, JW, and AD contributed to the conception and design of the study. YM and JW conducted the statistical analyses. AD performed the literature search and drafted the first version of the manuscript, with input from YM and JW. XL, JG, ME, SS, and QZ interpreted the data and provided critical revisions to the manuscript. YM, JW, and AD verified the underlying data. All authors made a significant contribution to finalising the manuscript and have read and approved the final version for publication.

## Data sharing statement

The data is not publicly available, but researchers can apply for access at: https://www.ukbiobank.ac.uk/enable-your-research/apply-for-access.

## Declaration of interests

Nothing to disclose.
