# Supplementary material for: Poor sleep health is associated with older brain age: the role of systemic inflammation
Source: eBioMedicine. 2025 Sep 30;120:105941. doi: 10.1016/j.ebiom.2025.105941 (PMC12571578; doi:10.1016/j.ebiom.2025.105941)
Supplement: Supplementary Materials [file mmc1.docx]

**Supplementary Materials**

**Figure S1—**Selection of the study sample.


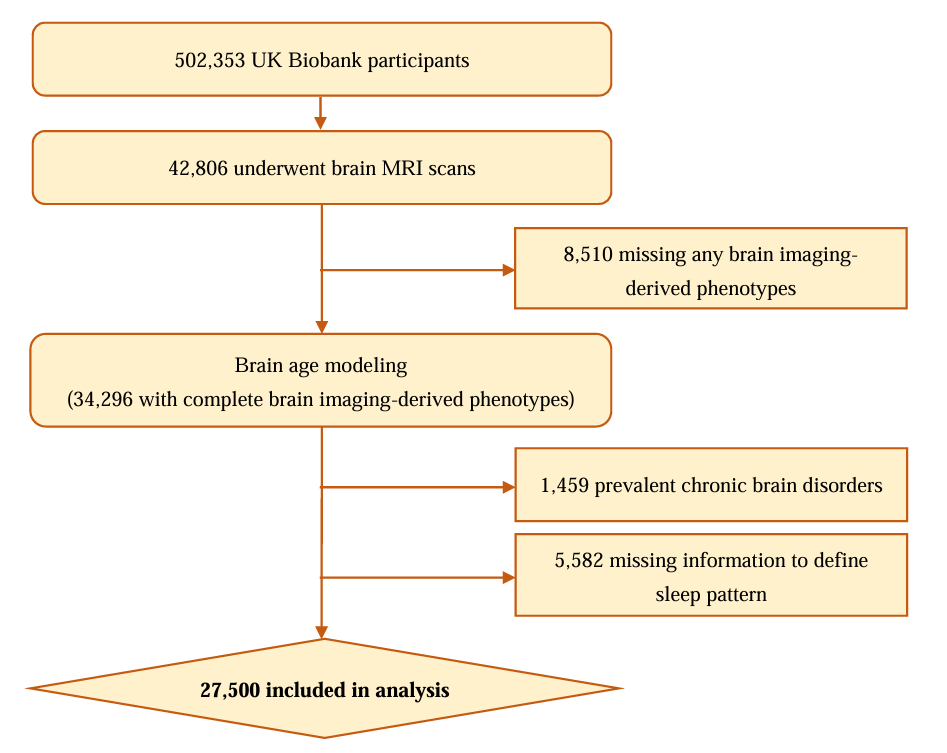


**Table S1—**Neurological disorders used as exclusion criteria for the neuroimaging subsample.

| Neurological disorder (self-reported) | Code (Field ID 20002 and 20003) |
| --- | --- |
| Parkinson’s disease | 1262 |
| Dementia or Alzheimer’s disease | 1263 |
| Chronic degenerative neurological | 1258 |
| Guillain-Barré syndrome | 1256 |
| Multiple Sclerosis | 1261 |
| Other demyelinating disease | 1397 |
| Stroke or ischemic stroke | 1081 |
| Brain cancer | 1032 |
| Brain haemorrhage | 1491 |
| Brain/intracranial abscess | 1245 |
| Cerebral aneurysm | 1425 |
| Cerebral palsy | 1433 |
| Encephalitis | 1246 |
| Epilepsy | 1264 |
| Head injury | 1266 |
| Infections of the nervous system | 1244 |
| Ischemic stroke | 1583 |
| Meningeal cancer | 1031 |
| Meningioma (benign) | 1659 |
| Meningitis | 1247 |
| Motor neuron disease | 1259 |
| Neurological injury/trauma | 1240 |
| Spina bifida | 1524 |
| Subdural hematoma | 1083 |
| Subarachnoid haemorrhage | 1086 |
| Transient ischemic attack | 1082 |

**APPENDIX I: Machine learning-based calculation of brain age**

A total of 1,079 imaging-derived phenotypes (IDPs) were derived from six brain MRI modalities **(Table S2)**. Brain age was estimated among all participants with complete data available for all 1,079 IDPs (n=34,296), according to the following procedure:

**Data Processing**. The model was initially trained and validated in a subset of healthy participants, as brain age is assumed to match chronological age in people who are disease-free and aging normally. From the entire sample of participants with complete brain MRI data (n=34,296), we excluded those with ICD-10 diagnoses, self-reported long-term illness, disability, or frailty (Field ID: 2188), and self-reported fair or poor health status (Field ID: 2178), yielding a sample of 4,355 healthy participants. These participants were randomly allocated in a 4:1 ratio to a training set (n=3,484) and a validation set (n=871).

Next, all 1,079 IDPs were standardized using Z-score conversion. IDPs in the training set were converted to Z-scores as follows: *standardized IDP_training set_ = (IDP_training set_ – mean[IDP]_training set_) / SD(IDP)_training set_.* IDPs in the validation/testing set converted to Z-scores based on the means and standard deviations of the IDPs in training set (a method to prevent data leakage): *IDP_validation/testing set_ = (IDP_validation/testing set_ – mean[IDP]_training set_) / SD(IDP)_training set_.*

**Model Tuning.** A total of nine machine learning models were trained for modeling brain age in the training set. These included Least Absolute Shrinkage and Selection Operator (LASSO) regression, eXtreme Gradient Boosting (XGBoost), and Support Vector Regression (SVR), which were combined with three possible feature selection strategies (no feature selection, FeatureWiz or Recursive Feature Elimination with Cross Validation [RFECV]). Bayesian optimization was performed to optimize the hyperparameters of all 9 models through 100 epochs utilizing 5-fold cross-validation and employing "neg_mean_absolute_error" (the negative of mean absolute error, where higher value means better performance) as the scoring rule. After selecting the best parameters for the 9 models through Bayesian optimization, models were subsequently applied the validation set.

**Model Evaluating.** Model performance was primarily evaluated using mean absolute error (MAE), in alignment with previous brain age studies, but we also assessed performance in terms of R-squared, Pearson’s r, mean square error and explained variance score. Ultimately, the LASSO model without feature selection achieved the lowest MAE (**Table S3**) and was therefore chosen to predict brain age for the entire sample.

In this model, 285 of the 1,079 IDPs contributed significantly to the brain age estimate (**Table S4**). The IDPs that contributed mostly strongly to the brain age estimate predominantly included total volumes and grey matter volumes of various brain regions from T1-weighted scans, fractional anisotropy in different brain regions from diffusion MRI scans, and hypointensities in different brain regions from T2* scans. Therefore, the brain age estimate can be understood as being reflective of a wide range of potential brain pathologies, including brain atrophy, white matter damage, and microbleeds.

**Correction for Age Bias.** Brain age models tend to overpredict brain age in younger subjects and underpredict brain age in older subjects, we corrected brain age estimates for age bias as follows: *brain age_corrected_ = [brain age_original_ – β/α]*, where coefficients *α* and *β* are the slope and intercept of: *brain age_training set_ = α*chronological age_training set_ + β*.

**Model Applying.** Brain-predicted age difference (BAG) represents the difference between an individual’s brain age and their chronological age and is calculated as follows: BAG = brain age – age_time of MRI_.

**Table S2—**UK Biobank brain MRI image acquisition protocols.

| Modality | Duration (minutes) | Voxel, Matrix | Key Parameters |
| --- | --- | --- | --- |
| T1 | 4:54 | 1×1×1 mm  208×256×256 | 3D MPRAGE, sagittal, R=2, TI/TR=880/2000 ms |
| T2 FLAIR | 5:52 | 1.05×1.0×1.0 mm  192×256×256 | FLAIR, 3D SPACE, sagittal, R=2, PF 7/8, fat sat, TI/TR=1800/5000 ms, elliptical |
| T2* | 2:34 | 0.8 × 0.8 × 3 mm  256 × 288 × 48 | 3D GRE, axial, R=2, PF 7/8 TE1/TE2/TR = 9.4/20/27 ms |
| Diffusion MRI | 7:08 | 2.0×2.0×2.0 mm  104×104×72 | MB=3, R=1, TE/TR=92/3600 ms, PF 6/8, fat sat, b=0 s/mm^2^ (5x + 3×phase-encoding reversed), b=1 000 s/mm^2^ (50×), b=2000 s/mm^2^ (50×) |
| Resting-state fMRI | 6:10 | 2.4 × 2.4 × 2.4 mm  88 × 88 × 64 | TE/TR = 39/735 ms, MB = 8, R = 1, flip angle 52°, fat sat |
| Task fMRI | 4:13 | 2.4 × 2.4 × 2.4 mm  88 × 88 × 64 | Acquisition same as rfMRI. Task is faces/shapes “emotion” task. |

Abbreviations: R, in-plane acceleration factor; MB, multiband factor; PF, partial Fourier.

All non-EPI scans are pre-scan normalized (on-scanner bias-field corrected). Gradient distortion correction is deselected on the scanner and applied in post-processing.

Table modified from Alfaro-Almagro et al. *Neuroimage* 2018 (doi.org/10.1016/j.neuroimage.2017.10.034)

**Table S3—**Performance comparison for nine candidate machine learning models for brain age calculation.

| Model | Feature Selector | MAE | R^2^ | *Pearson’s* r | MSE | Explained Variance Score |
| --- | --- | --- | --- | --- | --- | --- |
| **Validation set** |  |  |  |  |  |  |
| XGBoost | None | 3.411 | 0.650 | 0.806 | 18.300 | 0.651 |
| LASSO | None | 3.232 | 0.690 | 0.830 | 16.225 | 0.692 |
| SVR | None | 3.235 | 0.680 | 0.825 | 16.731 | 0.681 |
| XGBoost | FeatureWiz | 3.285 | 0.676 | 0.822 | 16.967 | 0.677 |
| LASSO | FeatureWiz | 3.301 | 0.677 | 0.823 | 16.903 | 0.679 |
| SVR | FeatureWiz | 3.283 | 0.677 | 0.823 | 16.898 | 0.677 |
| XGBoost | RFECV | 3.415 | 0.650 | 0.806 | 18.322 | 0.651 |
| LASSO | RFECV | 3.348 | 0.666 | 0.816 | 17.472 | 0.668 |
| SVR | RFECV | 3.412 | 0.645 | 0.803 | 18.539 | 0.648 |
| **Testing set** |  |  |  |  |  |  |
| XGBoost | None | 3.552 | 0.662 | 0.814 | 19.859 | 0.664 |
| LASSO | None | 3.416 | 0.675 | 0.822 | 19.118 | 0.676 |
| SVR | None | 3.455 | 0.680 | 0.825 | 18.811 | 0.682 |
| XGBoost | FeatureWiz | 3.526 | 0.667 | 0.817 | 19.577 | 0.669 |
| LASSO | FeatureWiz | 3.482 | 0.666 | 0.816 | 19.619 | 0.668 |
| SVR | FeatureWiz | 3.491 | 0.674 | 0.821 | 19.178 | 0.676 |
| XGBoost | RFECV | 3.545 | 0.663 | 0.814 | 19.820 | 0.665 |
| LASSO | RFECV | 3.525 | 0.657 | 0.811 | 20.155 | 0.659 |
| SVR | RFECV | 3.548 | 0.663 | 0.814 | 19.834 | 0.664 |

Abbreviation: LASSO, least absolute shrinkage and selection operator; MAE, mean absolute error; MSE, mean square error; RFECV, recursive feature elimination cross-validation; R^2^, R-squared; SVR, support vector regression; XGBoost, eXtreme Gradient Boosting.

**Table S4—**Coefficients for 285 IDPs that significantly contribute to brain age estimation in the LASSO regression without feature selection.

| Imaging-Derived Phenotype (IDP) | Coefficient |
| --- | --- |
| Volume of grey matter (normalized for head size) | -1.3516 |
| Weighted-mean ICVF in tract forceps minor | -0.7169 |
| Mean ISOVF in fornix on FA skeleton | 0.6877 |
| Volume of grey matter in Ventral Striatum (left) | -0.6099 |
| Mean FA in superior cerebellar peduncle on FA skeleton (right) | 0.5779 |
| Volume of brain stem + 4th ventricle | 0.5745 |
| Weighted-mean OD in tract anterior thalamic radiation (right) | -0.5529 |
| Mean L1 in anterior limb of internal capsule on FA skeleton (right) | 0.5410 |
| Volume of thalamus (right) | -0.5232 |
| Weighted-mean FA in tract forceps minor | -0.5113 |
| Mean FA in cerebral peduncle on FA skeleton (left) | -0.4964 |
| Volume of grey matter in Putamen (left) | 0.4557 |
| Mean L1 in middle cerebellar peduncle on FA skeleton | -0.4240 |
| Volume of grey matter in Insular Cortex (left) | 0.4107 |
| Volume of grey matter in VI Cerebellum (left) | -0.4004 |
| Volume of putamen (left) | -0.3795 |
| Median T2star in putamen (left) | -0.3773 |
| Volume of grey matter in IX Cerebellum (left) | 0.3740 |
| Mean L1 in anterior limb of internal capsule on FA skeleton (left) | 0.3556 |
| Mean MO in fornix cres+stria terminalis on FA skeleton (left) | -0.3367 |
| Weighted-mean OD in tract posterior thalamic radiation (right) | -0.3219 |
| Mean ICVF in superior longitudinal fasciculus on FA skeleton (right) | 0.3088 |
| Mean MO in fornix on FA skeleton | 0.3075 |
| Weighted-mean ISOVF in tract uncinate fasciculus (left) | 0.2999 |
| Volume of grey matter in Frontal Operculum Cortex (right) | -0.2969 |
| Mean L3 in posterior thalamic radiation on FA skeleton (right) | 0.2762 |
| Volume of thalamus (left) | -0.2757 |
| Mean L2 in fornix cres+stria terminalis on FA skeleton (left) | 0.2661 |
| Mean OD in posterior limb of internal capsule on FA skeleton (right) | 0.2650 |
| Mean FA in body of corpus callosum on FA skeleton | 0.2633 |
| Weighted-mean MO in tract acoustic radiation (left) | -0.2515 |
| Volume of grey matter in Heschl's Gyrus (includes H1 and H2) (right) | -0.2482 |
| Mean L1 in genu of corpus callosum on FA skeleton | 0.2473 |
| Mean L2 in splenium of corpus callosum on FA skeleton | -0.2430 |
| Weighted-mean ISOVF in tract forceps minor | -0.2387 |
| Volume of putamen (right) | -0.2383 |
| Mean OD in anterior limb of internal capsule on FA skeleton (right) | 0.2364 |
| Weighted-mean ISOVF in tract superior thalamic radiation (right) | 0.2271 |
| Volume of grey matter in Lateral Occipital Cortex, inferior division (left) | 0.2193 |
| Mean ICVF in body of corpus callosum on FA skeleton | 0.2128 |
| rfMRI partial correlation matrix, dimension 25 (element 180) | 0.2090 |
| Mean ICVF in tapetum on FA skeleton (right) | -0.2087 |
| Median z-statistic (in group-defined amygdala activation mask) for faces-shapes contrast | -0.2082 |
| Mean OD in pontine crossing tract on FA skeleton | 0.2078 |
| Mean L3 in retrolenticular part of internal capsule on FA skeleton (left) | -0.2076 |
| Weighted-mean ISOVF in tract superior longitudinal fasciculus (right) | 0.2068 |
| Mean ISOVF in superior corona radiata on FA skeleton (right) | 0.1990 |
| rfMRI partial correlation matrix, dimension 25 (element 127) | 0.1965 |
| Mean L1 in sagittal stratum on FA skeleton (right) | -0.1930 |
| Mean ICVF in fornix cres+stria terminalis on FA skeleton (right) | -0.1921 |
| Weighted-mean L3 in tract medial lemniscus (right) | -0.1885 |
| rfMRI partial correlation matrix, dimension 25 (element 44) | -0.1877 |
| Volume of grey matter in Inferior Temporal Gyrus, temporooccipital part (left) | 0.1851 |
| Mean ICVF in retrolenticular part of internal capsule on FA skeleton (right) | 0.1833 |
| Mean FA in superior cerebellar peduncle on FA skeleton (left) | 0.1828 |
| rfMRI partial correlation matrix, dimension 25 (element 172) | 0.1826 |
| Weighted-mean L1 in tract parahippocampal part of cingulum (left) | -0.1823 |
| Weighted-mean ICVF in tract superior longitudinal fasciculus (right) | 0.1801 |
| Mean OD in superior cerebellar peduncle on FA skeleton (left) | -0.1790 |
| Volume of grey matter in Frontal Orbital Cortex (left) | -0.1696 |
| Volume of grey matter in X Cerebellum (left) | -0.1652 |
| Volume of grey matter in Paracingulate Gyrus (right) | -0.1651 |
| Weighted-mean L1 in tract corticospinal tract (left) | -0.1642 |
| Mean ICVF in medial lemniscus on FA skeleton (left) | 0.1599 |
| rfMRI partial correlation matrix, dimension 25 (element 22) | 0.1593 |
| Volume of grey matter in Hippocampus (left) | -0.1592 |
| Weighted-mean FA in tract middle cerebellar peduncle | -0.1569 |
| Mean ICVF in corticospinal tract on FA skeleton (right) | 0.1564 |
| 90th percentile of BOLD effect (in group-defined mask) for faces-shapes contrast | -0.1537 |
| Volume of grey matter in Crus II Cerebellum (vermis) | 0.1511 |
| rfMRI partial correlation matrix, dimension 25 (element 96) | 0.1498 |
| Weighted-mean ICVF in tract middle cerebellar peduncle | -0.1496 |
| Weighted-mean OD in tract anterior thalamic radiation (left) | -0.1485 |
| Mean L1 in superior corona radiata on FA skeleton (left) | 0.1481 |
| rfMRI partial correlation matrix, dimension 25 (element 151) | -0.1473 |
| rfMRI partial correlation matrix, dimension 25 (element 131) | -0.1440 |
| Mean L1 in superior longitudinal fasciculus on FA skeleton (left) | -0.1422 |
| Mean ISOVF in cingulum cingulate gyrus on FA skeleton (right) | -0.1402 |
| rfMRI partial correlation matrix, dimension 25 (element 89) | 0.1397 |
| Mean ICVF in corticospinal tract on FA skeleton (left) | 0.1388 |
| Volume of grey matter in Frontal Operculum Cortex (left) | -0.1377 |
| rfMRI partial correlation matrix, dimension 25 (element 46) | -0.1336 |
| Volume of grey matter in X Cerebellum (right) | -0.1322 |
| Mean FA in fornix on FA skeleton | -0.1321 |
| Median T2star in thalamus (left) | 0.1312 |
| Mean FA in corticospinal tract on FA skeleton (right) | 0.1299 |
| Weighted-mean MO in tract acoustic radiation (right) | -0.1284 |
| Volume of grey matter in Lingual Gyrus (right) | -0.1282 |
| Mean L1 in cerebral peduncle on FA skeleton (right) | -0.1270 |
| rfMRI partial correlation matrix, dimension 25 (element 146) | 0.1266 |
| rfMRI partial correlation matrix, dimension 25 (element 135) | -0.1256 |
| Weighted-mean L1 in tract corticospinal tract (right) | -0.1253 |
| Volume of grey matter in Angular Gyrus (left) | 0.1231 |
| Mean L2 in cingulum cingulate gyrus on FA skeleton (right) | -0.1207 |
| Volume of grey matter in Supramarginal Gyrus, anterior division (right) | 0.1195 |
| Volume of grey matter in Crus I Cerebellum (vermis) | -0.1187 |
| Volume of grey matter in Planum Polare (left) | -0.1185 |
| Volume of grey matter in Pallidum (left) | 0.1175 |
| Volume of grey matter in Planum Polare (right) | -0.1166 |
| Weighted-mean MO in tract forceps major | -0.1160 |
| Mean ICVF in posterior corona radiata on FA skeleton (left) | 0.1153 |
| Weighted-mean ICVF in tract anterior thalamic radiation (right) | -0.1150 |
| Volume of grey matter in Frontal Orbital Cortex (right) | -0.1143 |
| rfMRI partial correlation matrix, dimension 25 (element 159) | 0.1122 |
| rfMRI partial correlation matrix, dimension 25 (element 161) | -0.1119 |
| Weighted-mean OD in tract superior longitudinal fasciculus (left) | 0.1116 |
| Weighted-mean L2 in tract posterior thalamic radiation (right) | 0.1114 |
| rfMRI partial correlation matrix, dimension 25 (element 49) | -0.1111 |
| Weighted-mean ICVF in tract corticospinal tract (right) | 0.1107 |
| rfMRI partial correlation matrix, dimension 25 (element 202) | 0.1087 |
| Weighted-mean L1 in tract acoustic radiation (left) | -0.1083 |
| Mean MD in cingulum cingulate gyrus on FA skeleton (right) | -0.1082 |
| Volume of grey matter in Frontal Medial Cortex (right) | -0.1079 |
| Mean L1 in superior corona radiata on FA skeleton (right) | 0.1078 |
| rfMRI partial correlation matrix, dimension 25 (element 124) | -0.1065 |
| Mean FA in middle cerebellar peduncle on FA skeleton | -0.1061 |
| Volume of grey matter in Occipital Fusiform Gyrus (left) | 0.1054 |
| 90th percentile of z-statistic (in group-defined amygdala activation mask) for faces-shapes contrast | -0.1052 |
| rfMRI partial correlation matrix, dimension 25 (element 58) | 0.1025 |
| Mean ISOVF in inferior cerebellar peduncle on FA skeleton (left) | -0.1009 |
| Mean MO in medial lemniscus on FA skeleton (right) | 0.1004 |
| Mean FA in fornix cres+stria terminalis on FA skeleton (right) | -0.0995 |
| Volume of grey matter in Thalamus (right) | 0.0991 |
| Mean ICVF in posterior thalamic radiation on FA skeleton (left) | -0.0989 |
| Weighted-mean OD in tract posterior thalamic radiation (left) | -0.0978 |
| rfMRI partial correlation matrix, dimension 25 (element 66) | -0.0954 |
| rfMRI partial correlation matrix, dimension 25 (element 60) | -0.0949 |
| Mean OD in superior cerebellar peduncle on FA skeleton (right) | -0.0947 |
| Mean MO in medial lemniscus on FA skeleton (left) | 0.0945 |
| rfMRI partial correlation matrix, dimension 25 (element 155) | 0.0934 |
| rfMRI partial correlation matrix, dimension 25 (element 144) | -0.0924 |
| Weighted-mean MO in tract cingulate gyrus part of cingulum (left) | -0.0910 |
| Volume of grey matter in Inferior Temporal Gyrus, posterior division (right) | -0.0895 |
| rfMRI partial correlation matrix, dimension 25 (element 156) | 0.0885 |
| Weighted-mean MD in tract corticospinal tract (right) | -0.0885 |
| rfMRI partial correlation matrix, dimension 25 (element 130) | -0.0881 |
| Volume of grey matter in Crus II Cerebellum (right) | -0.0856 |
| Volume of grey matter in VIIIb Cerebellum (vermis) | 0.0850 |
| Median T2star in thalamus (right) | 0.0843 |
| Volume of grey matter in Supramarginal Gyrus, posterior division (right) | -0.0840 |
| rfMRI partial correlation matrix, dimension 25 (element 103) | -0.0840 |
| Mean MD in uncinate fasciculus on FA skeleton (right) | 0.0835 |
| Mean ICVF in superior fronto-occipital fasciculus on FA skeleton (right) | -0.0832 |
| rfMRI partial correlation matrix, dimension 25 (element 204) | 0.0819 |
| Weighted-mean ISOVF in tract middle cerebellar peduncle | -0.0816 |
| Mean MO in fornix cres+stria terminalis on FA skeleton (right) | -0.0807 |
| Mean L1 in superior fronto-occipital fasciculus on FA skeleton (left) | 0.0799 |
| Mean MO in external capsule on FA skeleton (right) | -0.0797 |
| Mean OD in fornix on FA skeleton | -0.0796 |
| rfMRI partial correlation matrix, dimension 25 (element 25) | -0.0790 |
| rfMRI partial correlation matrix, dimension 25 (element 121) | -0.0781 |
| Weighted-mean ISOVF in tract parahippocampal part of cingulum (right) | 0.0780 |
| rfMRI partial correlation matrix, dimension 25 (element 97) | 0.0779 |
| rfMRI partial correlation matrix, dimension 25 (element 125) | 0.0772 |
| Mean ICVF in inferior cerebellar peduncle on FA skeleton (left) | -0.0749 |
| Volume of grey matter in Planum Temporale (left) | -0.0745 |
| Mean FA in inferior cerebellar peduncle on FA skeleton (left) | -0.0728 |
| Weighted-mean MO in tract posterior thalamic radiation (left) | -0.0724 |
| Mean OD in posterior thalamic radiation on FA skeleton (right) | 0.0715 |
| rfMRI partial correlation matrix, dimension 25 (element 84) | -0.0707 |
| rfMRI partial correlation matrix, dimension 25 (element 55) | 0.0682 |
| rfMRI partial correlation matrix, dimension 25 (element 142) | -0.0678 |
| 90th percentile of BOLD effect (in group-defined amygdala activation mask) for faces-shapes contrast | 0.0666 |
| Mean OD in posterior limb of internal capsule on FA skeleton (left) | 0.0662 |
| Mean FA in anterior limb of internal capsule on FA skeleton (left) | 0.0661 |
| rfMRI partial correlation matrix, dimension 25 (element 101) | 0.0657 |
| rfMRI partial correlation matrix, dimension 25 (element 31) | 0.0647 |
| rfMRI partial correlation matrix, dimension 25 (element 205) | 0.0644 |
| rfMRI partial correlation matrix, dimension 25 (element 35) | -0.0634 |
| Weighted-mean L1 in tract superior thalamic radiation (right) | 0.0622 |
| Mean MD in retrolenticular part of internal capsule on FA skeleton (right) | -0.0617 |
| Weighted-mean MO in tract medial lemniscus (left) | -0.0614 |
| Mean OD in external capsule on FA skeleton (right) | 0.0612 |
| Mean ISOVF in posterior limb of internal capsule on FA skeleton (left) | -0.0589 |
| Median T2star in accumbens (left) | -0.0570 |
| Weighted-mean ISOVF in tract anterior thalamic radiation (left) | -0.0569 |
| rfMRI partial correlation matrix, dimension 25 (element 169) | 0.0560 |
| Mean MO in inferior cerebellar peduncle on FA skeleton (right) | -0.0556 |
| rfMRI partial correlation matrix, dimension 25 (element 92) | -0.0552 |
| Mean OD in cingulum cingulate gyrus on FA skeleton (left) | -0.0534 |
| rfMRI partial correlation matrix, dimension 25 (element 94) | 0.0528 |
| Mean OD in uncinate fasciculus on FA skeleton (right) | 0.0502 |
| Volume of grey matter in Cingulate Gyrus, anterior division (left) | -0.0490 |
| Weighted-mean OD in tract uncinate fasciculus (right) | -0.0490 |
| Weighted-mean OD in tract parahippocampal part of cingulum (right) | 0.0468 |
| Mean OD in anterior corona radiata on FA skeleton (right) | -0.0465 |
| Weighted-mean ISOVF in tract posterior thalamic radiation (right) | 0.0450 |
| rfMRI partial correlation matrix, dimension 25 (element 21) | -0.0448 |
| rfMRI partial correlation matrix, dimension 25 (element 194) | 0.0446 |
| Volume of grey matter in VI Cerebellum (right) | -0.0435 |
| rfMRI partial correlation matrix, dimension 25 (element 128) | 0.0433 |
| Mean ISOVF in cingulum hippocampus on FA skeleton (left) | 0.0425 |
| Mean OD in corticospinal tract on FA skeleton (left) | -0.0416 |
| rfMRI partial correlation matrix, dimension 25 (element 143) | 0.0412 |
| Mean MD in cingulum hippocampus on FA skeleton (right) | -0.0410 |
| rfMRI partial correlation matrix, dimension 25 (element 39) | -0.0407 |
| Volume of amygdala (left) | 0.0403 |
| rfMRI partial correlation matrix, dimension 25 (element 51) | -0.0399 |
| Mean L2 in posterior corona radiata on FA skeleton (left) | -0.0396 |
| rfMRI partial correlation matrix, dimension 25 (element 210) | 0.0390 |
| rfMRI partial correlation matrix, dimension 25 (element 95) | -0.0380 |
| Mean L2 in fornix cres+stria terminalis on FA skeleton (right) | 0.0377 |
| rfMRI partial correlation matrix, dimension 25 (element 50) | -0.0375 |
| rfMRI partial correlation matrix, dimension 25 (element 15) | 0.0372 |
| Weighted-mean MO in tract superior longitudinal fasciculus (right) | -0.0366 |
| Weighted-mean MD in tract superior thalamic radiation (left) | 0.0364 |
| Mean OD in cerebral peduncle on FA skeleton (right) | 0.0360 |
| Mean ISOVF in anterior limb of internal capsule on FA skeleton (right) | 0.0348 |
| Volume of grey matter in Subcallosal Cortex (right) | 0.0343 |
| Volume of grey matter in VIIb Cerebellum (vermis) | -0.0337 |
| Volume of grey matter in Occipital Pole (left) | -0.0329 |
| rfMRI partial correlation matrix, dimension 25 (element 189) | 0.0328 |
| Median T2star in pallidum (right) | 0.0326 |
| rfMRI partial correlation matrix, dimension 25 (element 183) | 0.0325 |
| Mean MD in cingulum cingulate gyrus on FA skeleton (left) | -0.0322 |
| Mean L1 in pontine crossing tract on FA skeleton | -0.0321 |
| rfMRI partial correlation matrix, dimension 25 (element 140) | -0.0305 |
| rfMRI partial correlation matrix, dimension 25 (element 75) | 0.0302 |
| rfMRI partial correlation matrix, dimension 25 (element 5) | 0.0301 |
| Volume of grey matter in Brain-Stem | -0.0301 |
| rfMRI partial correlation matrix, dimension 25 (element 176) | 0.0294 |
| rfMRI partial correlation matrix, dimension 25 (element 85) | -0.0294 |
| Mean L3 in posterior limb of internal capsule on FA skeleton (left) | -0.0290 |
| rfMRI partial correlation matrix, dimension 25 (element 82) | -0.0284 |
| rfMRI partial correlation matrix, dimension 25 (element 123) | -0.0283 |
| 90th percentile of BOLD effect (in group-defined mask) for shapes activation | 0.0279 |
| Volume of grey matter in Lingual Gyrus (left) | -0.0278 |
| rfMRI partial correlation matrix, dimension 25 (element 26) | 0.0269 |
| Mean ICVF in superior cerebellar peduncle on FA skeleton (right) | 0.0266 |
| Weighted-mean OD in tract inferior longitudinal fasciculus (right) | 0.0264 |
| rfMRI partial correlation matrix, dimension 25 (element 141) | 0.0259 |
| rfMRI partial correlation matrix, dimension 25 (element 106) | -0.0257 |
| rfMRI partial correlation matrix, dimension 25 (element 16) | 0.0245 |
| rfMRI partial correlation matrix, dimension 25 (element 112) | 0.0243 |
| Volume of grey matter in I-IV Cerebellum (right) | -0.0236 |
| rfMRI partial correlation matrix, dimension 25 (element 116) | -0.0235 |
| Weighted-mean ISOVF in tract anterior thalamic radiation (right) | -0.0232 |
| Median BOLD effect (in group-defined mask) for faces activation | 0.0229 |
| rfMRI partial correlation matrix, dimension 25 (element 64) | -0.0224 |
| Volume of grey matter in IX Cerebellum (right) | 0.0214 |
| Volume of grey matter in Temporal Fusiform Cortex, posterior division (left) | -0.0212 |
| rfMRI partial correlation matrix, dimension 25 (element 196) | -0.0201 |
| Weighted-mean MO in tract posterior thalamic radiation (right) | -0.0199 |
| rfMRI partial correlation matrix, dimension 25 (element 29) | 0.0190 |
| rfMRI partial correlation matrix, dimension 25 (element 71) | 0.0189 |
| Mean L2 in posterior limb of internal capsule on FA skeleton (right) | 0.0187 |
| rfMRI partial correlation matrix, dimension 25 (element 56) | -0.0180 |
| rfMRI partial correlation matrix, dimension 25 (element 17) | -0.0175 |
| Volume of grey matter in Inferior Temporal Gyrus, anterior division (right) | -0.0168 |
| Volume of grey matter in Juxtapositional Lobule Cortex (formerly Supplementary Motor Cortex) (left) | -0.0155 |
| Mean L2 in sagittal stratum on FA skeleton (left) | -0.0150 |
| rfMRI partial correlation matrix, dimension 25 (element 164) | 0.0149 |
| rfMRI partial correlation matrix, dimension 25 (element 133) | 0.0144 |
| Volume of grey matter in Lateral Occipital Cortex, superior division (right) | -0.0140 |
| rfMRI partial correlation matrix, dimension 25 (element 107) | 0.0136 |
| Volume of grey matter in Cuneal Cortex (right) | 0.0131 |
| Mean L1 in cerebral peduncle on FA skeleton (left) | -0.0126 |
| Volume of grey matter in Superior Temporal Gyrus, posterior division (left) | -0.0125 |
| Volume of grey matter in Supramarginal Gyrus, anterior division (left) | 0.0123 |
| rfMRI partial correlation matrix, dimension 25 (element 113) | -0.0123 |
| Mean L2 in retrolenticular part of internal capsule on FA skeleton (right) | -0.0121 |
| rfMRI partial correlation matrix, dimension 25 (element 80) | 0.0118 |
| Mean OD in retrolenticular part of internal capsule on FA skeleton (left) | 0.0109 |
| Mean MO in cerebral peduncle on FA skeleton (left) | -0.0108 |
| Volume of grey matter in Superior Parietal Lobule (left) | -0.0106 |
| rfMRI partial correlation matrix, dimension 25 (element 98) | 0.0094 |
| Weighted-mean OD in tract cingulate gyrus part of cingulum (left) | 0.0089 |
| Mean L1 in uncinate fasciculus on FA skeleton (right) | 0.0084 |
| rfMRI partial correlation matrix, dimension 25 (element 7) | -0.0081 |
| Weighted-mean OD in tract uncinate fasciculus (left) | 0.0079 |
| rfMRI partial correlation matrix, dimension 25 (element 197) | -0.0077 |
| Median T2star in pallidum (left) | 0.0067 |
| rfMRI partial correlation matrix, dimension 25 (element 190) | 0.0066 |
| rfMRI partial correlation matrix, dimension 25 (element 206) | 0.0063 |
| Weighted-mean MO in tract forceps minor | -0.0062 |
| Volume of grey matter in Frontal Pole (left) | -0.0060 |
| Mean L3 in posterior thalamic radiation on FA skeleton (left) | 0.0059 |
| rfMRI partial correlation matrix, dimension 25 (element 136) | -0.0057 |
| rfMRI partial correlation matrix, dimension 25 (element 179) | 0.0053 |
| Mean MO in cingulum hippocampus on FA skeleton (right) | 0.0044 |
| Mean OD in superior fronto-occipital fasciculus on FA skeleton (left) | -0.0034 |
| Weighted-mean OD in tract acoustic radiation (left) | 0.0023 |
| rfMRI partial correlation matrix, dimension 25 (element 186) | 0.0019 |
| Volume of grey matter in VI Cerebellum (vermis) | -0.0013 |
| rfMRI partial correlation matrix, dimension 25 (element 87) | -0.0008 |

**Table S5—**Baseline characteristics of the study populations by healthy sleep score.

| Characteristics | Healthy sleep score | | | | | P-value |
| --- | --- | --- | --- | --- | --- | --- |
|  | 0-1  (n = 898) | 2  (n =4,870) | 3  (n = 10,413) | 4  (n = 9,006) | 5  (n = 2,313) |  |
| Age at baseline (year) | 54.63 ± 7.18 | 55.05 ± 7.33 | 54.98 ± 7.36 | 54.53 ± 7.59 | 53.24 ± 7.92 | <0.001 |
| Age at MRI assessment (year) | 63.42 ± 7.38 | 63.88 ± 7.51 | 63.89 ± 7.49 | 63.44 ± 7.73 | 62.23 ± 8.06 | <0.001 |
| Female | 406 (45.21) | 2,360 (48.46) | 5,635 (54.12) | 5,175 (57.46) | 1,285 (55.56) | <0.001 |
| Race-White | 69 (7.69) | 358 (7.36) | 767 (7.38) | 638 (7.10) | 160 (6.93) | 0.863 |
| Townsend deprivation index | -2.41 (-3.71, 0.02) | -2.50 (-3.83, -0.22) | -2.68 (-3.91, -0.64) | -2.71 (-3.93, -0.76) | -2.70 (-3.90, -0.71) | <0.001 |
| Education (college) | 345 (38.50) | 2,105 (43.33) | 4,724 (45.46) | 4,482 (49.89) | 1,173 (50.78) | <0.001 |
| BMI (kg/m^2^) | 28.37 ± 4.98 | 27.54 ± 4.43 | 26.53 ± 4.10 | 25.83 ± 3.87 | 25.59 ± 3.82 | <0.001 |
| Healthy lifestyle | 121 (16.37) | 745 (17.78) | 1,793 (20.08) | 1,653 (21.41) | 488 (24.77) | <0.001 |
| Non-smoking | 456 (50.78) | 2,704 (55.67) | 6,178 (59.44) | 5,741 (63.85) | 1,569 (67.89) | <0.001 |
| No heavy drinking | 60 (7.73) | 228 (5.24) | 576 (6.25) | 491 (6.20) | 166 (8.25) | <0.001 |
| Regular physical activity | 560 (65.34) | 3,291 (70.11) | 7,513 (74.36) | 6,742 (76.80) | 1,830 (80.87) | <0.001 |
| High level of social contact | 352 (39.29) | 1,896 (39.04) | 4,043 (38.90) | 3,524 (39.16) | 920 (39.79) | 0.954 |
| Cardiometabolic disease | 479 (53.34) | 2,532 (51.99) | 4,826 (46.35) | 3,816 (42.37) | 864 (37.35) | <0.001 |
| Coronary artery disease | 37 (4.12) | 157 (3.22) | 302 (2.90) | 191 (2.12) | 41 (1.77) | <0.001 |
| Atrial fibrillation | 11 (1.22) | 54 (1.11) | 115 (1.10) | 76 (0.84) | 17 (0.73) | 0.194 |
| Heart failure | 0 (0.00) | 5 (0.10) | 20 (0.19) | 9 (0.10) | 2 (0.09) | 0.243 |
| Hypertension | 462 (51.45) | 2,457 (50.45) | 4,659 (44.74) | 3,681 (40.87) | 833 (36.01) | <0.001 |
| Diabetes | 43 (4.79) | 166 (3.41) | 262 (2.52) | 195 (2.17) | 39 (1.69) | <0.001 |
| *APOE* ɛ4 carriers | 210 (27.45) | 1,091 (26.94) | 2,464 (27.94) | 2,105 (27.68) | 530 (27.15) | 0.806 |
| INFLA-score | 0.00 (-5.00, 4.00) | -1.00 (-5.00, 3.00) | -1.00 (-5.00, 3.00) | -2.00 (-6.00, 2.00) | -2.00 (-6.00, 2.00) | <0.001 |

Abbreviations: BMI = body mass index; *APOE* = *apolipoprotein* E.

Missing data: Education = 61 (0.2%); Race = 54 (0.2%); Townsend deprivation index = 23 (0.1%); Smoking = 49 (0.2%); Alcohol consumption = 5 (0.02%); Regular physical activity = 804 (2.9%); Regular social contact =44 (0.2%); *APOE* ɛ4 = 4,311 (15.7%); INFLA-score =3,040 (11.1%).

**Table S6—**Associations between individual sleep characteristics and brain age gap (BAG).

| Sleep characteristics | No. of participants | LS Mean ± SE |  | BAG |  |
| --- | --- | --- | --- | --- | --- |
|  |  |  |  | β (95% CI) | *P*-value |
| **Chronotype** |  |  |  |  |  |
| “Morning,” “more morning than evening” | 17,094 | 0.39 ± 0.04 |  | Reference | - |
| “Evening,” “more evening than morning” | 10,046 | 0.64 ± 0.05 |  | **0.23 (0.09, 0.36)** | **0.001** |
| **Sleep duration** |  |  |  |  |  |
| 7–8 h/day | 20,065 | 0.39 ± 0.32 |  | Reference | - |
| <7 or ≥8 h/day | 7,435 | 0.73 ± 0.06 |  | **0.26 (0.12, 0.41)** | **<0.001** |
| **Insomnia** |  |  |  |  |  |
| “Never/rarely” | 19,871 | 0.53 ± 0.06 |  | Reference | - |
| “Sometimes” or “usually” | 7,629 | 0.46 ± 0.04 |  | 0.09 (-0.05, 0.24) | 0.194 |
| **Snoring** |  |  |  |  |  |
| No | 17,67 | 0.36 ±0.04 |  | Reference | - |
| Yes | 9,803 | 0.69 ± 0.04 |  | **0.14 (0.01, 0.28)** | **0.042** |
| **Subjective daytime sleepiness** | |  |  |  |  |
| “Never/rarely” | 26,948 | 0.48 ± 0.03 |  | Reference | - |
| “Sometimes,” “often,” or “all the time” | 552 | 0.62 ± 0.24 |  | 0.07 (-0.40, 0.54) | 0.280 |

Models were adjusted for age, sex, education, race, socioeconomic status, healthy lifestyle (smoking status, alcohol intake, physical activity, and social contact), cardiometabolic disease, and brain MRI assessment centre.

**Table S7—**Subgroup analysis for the associations between sleep pattern and brain age gap (BAG).

| Sleep pattern |  | Subgroup |  |  |  | Subgroup |  |  | P for interaction |
| --- | --- | --- | --- | --- | --- | --- | --- | --- | --- |
|  |  | No. of participants | β (95% CI) | *P*-value |  | No. of participants | β (95% CI) | *P*-value |  |
|  |  | ***Age <60 years* (n = 19,005)** | | |  | ***Age ≥ 60 years* (n = 8,495)** | | |  |
| Healthy |  | 7,955 | Reference | - |  | 3,364 | Reference | - | - |
| Intermediate |  | 10,411 | 0.17 (0.02, 0.33) | 0.028 |  | 4,872 | 0.39 (0.14, 0.65) | 0.003 | 0.065 |
| Poor |  | 639 | 0.72 (0.28, 1.15) | 0.001 |  | 259 | -0.05 (-0.78, 0.69 | 0.904 | 0.142 |
| Healthy sleep score  (per 1-unit decrease) |  |  | 0.14 (0.06, 0.22) | <0.001 |  |  | 0.11 (-0.02, 0.25) | 0.094 | 0.693 |
|  |  | ***Female* (n = 14,861)** | | | | ***Male* (n = 12,639)** | | |  |
| Healthy |  | 6,460 | Reference | - |  | 4,859 | Reference | - | - |
| Intermediate |  | 7,995 | 0.13 (-0.04, 0.30) | 0.145 |  | 7,288 | 0.37 (0.17, 0.57) | 0.042 | 0.036 |
| Poor |  | 406 | 0.47 (-0.08, 1.01) | 0.092 |  | 492 | 0.55 (0.02, 1.08) | <0.001 | 0.651 |
| Healthy sleep score  (per 1-unit decrease) |  |  | 0.08 (-0.02, 0.16) | 0.150 |  |  | 0.20 (0.10, 0.30) | <0.001 | 0.023 |
|  |  | ***APOE ɛ4 non-carriers* (n = 16,789)** | | |  | ***APOE ɛ4 carriers* (n = 6,400)** | | |  |
| Healthy |  | 6,922 | Reference | - |  | 2,635 | Reference | - | - |
| Intermediate |  | 9,312 | 0.25 (0.08, 0.42) | 0.049 |  | 3,555 | 0.25 (-0.03, 0.53) | 0.262 | 0.964 |
| Poor |  | 555 | 0.47 (0.01, 0.95) | 0.003 |  | 210 | 0.46 (-0.35, 1.27) | 0.083 | 0.855 |
| Healthy sleep score  (per 1-unit decrease) |  |  | 0.13 (0.04, 0.21) | 0.004 |  |  | 0.18 (0.03, 0.32) | 0.019 | 0.670 |

Models included age, sex, education, race, socioeconomic status, healthy lifestyle (smoking status, alcohol intake, physical activity, and social contact), and cardiometabolic disease.

**Figure S2—**Distribution of INFLA-score by sleep pattern.


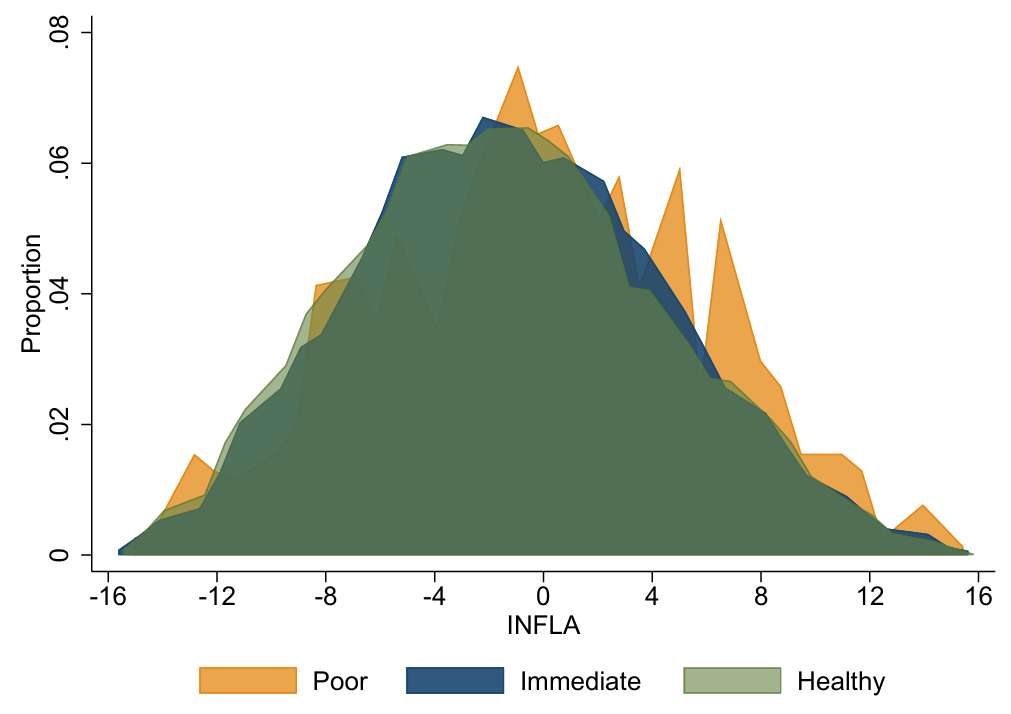


**Table S8—**Associations between healthy sleep pattern and brain age gap (BAG): using inverse probability weighted regression.

|  | No. of participants | LS Mean ± SE |  | BAG |  |
| --- | --- | --- | --- | --- | --- |
|  |  |  |  | β (95% CI) | *P*-value |
| Healthy sleep score  (per 1-unit decrease) | 27,500 | 0.48 ± 0.03 |  | **0.19 (0.12, 0.26)** | **<0.001** |
| 5 | 2,313 | 0.21 ± 0.11 |  | Reference | - |
| 4 | 9,006 | 0.27 ± 0.06 |  | 0.02 (-0.23, 0.27) | 0.883 |
| 3 | 10,413 | 0.57 ± 0.05 |  | 0.29 (0.04, 0.74) | 0.097 |
| 2 | 4,870 | 0.73 ± 0.08 |  | **0.47 (0.20, 0.74)** | **0.001** |
| 0-1 | 898 | 0.99 ± 0.18 |  | **0.71 (0.26, 1.15)** | **0.002** |
| Sleep pattern |  |  |  |  |  |
| Healthy | 11,319 | 0.26 ± 0.05 |  | Reference | - |
| Intermediate | 15,283 | 0.62 ± 0.04 |  | **0.33 (0.20, 0.47)** | **<0.001** |
| Poor | 898 | 0.99 ± 0.18 |  | **0.69 (0.30, 1.08)** | **0.001** |

Weights for the regressions were calculated using the inverse probability weighting method considering age, sex, education, race, socioeconomic status, healthy lifestyle (smoking status, alcohol intake, physical activity, and social contact), and cardiometabolic disease.

**Table S9—**Associations between healthy sleep pattern and brain age gap (BAG): after multiple imputation of covariates.

|  | No. of participants | LS Mean ± SE |  | BAG |  |
| --- | --- | --- | --- | --- | --- |
|  |  |  |  | β (95% CI) | *P*-value |
| Healthy sleep score  (per 1-unit decrease) | 27,500 | 0.49 ± 0.03 |  | **0.13 (0.06, 0.19)** | **<0.001** |
| 5 | 2,313 | 0.21 ± 0.11 |  | Reference | - |
| 4 | 9,006 | 0.27 ± 0.06 |  | 0.01 (-0.22, 0.23) | 0.969 |
| 3 | 10,413 | 0.57 ± 0.05 |  | 0.21 (-0.02, 0.43) | 0.074 |
| 2 | 4,870 | 0.73 ± 0.08 |  | **0.29 (0.04, 0.53)** | **0.024** |
| 0-1 | 898 | 0.99 ± 0.18 |  | **0.46 (0.07, 0.84)** | **0.021** |
| Sleep pattern |  |  |  |  |  |
| Healthy | 11,319 | 0.26 ± 0.05 |  | Reference | - |
| Intermediate | 15,283 | 0.62 ± 0.04 |  | **0.23 (0.11, 0.35)** | **<0.001** |
| Poor | 898 | 0.99 ± 0.18 |  | **0.45 (0.11, 0.79)** | **0.009** |

Models were adjusted for age, sex, education, race, socioeconomic status, healthy lifestyle (smoking status, alcohol intake, physical activity, and social contact), and cardiometabolic disease.

Missing values for covariates were imputed using multiple imputation by chained equations (MICE). A total of 10 imputed datasets were generated, and estimates were pooled using Stata’s “mi estimate” command, which combines estimates using within- and between-imputation variances.

**Table S10—**Associations between healthy sleep pattern and brain age gap (BAG): excluding 470 participants with baseline sleep disorders.

|  | No. of participants | LS Mean ± SE |  | BAG |  |
| --- | --- | --- | --- | --- | --- |
|  |  |  |  | β (95% CI) | *P*-value |
| Healthy sleep score  (per 1-unit decrease) | 27,030 | 0.40 ± 0.03 |  | **0.13 (0.07, 0.20)** | **<0.001** |
| 5 | 2,296 | 0.21 ± 0.11 |  | Reference | - |
| 4 | 8,901 | 0.26 ± 0.06 |  | 0.01 (-0.24, 0.25) | 0.968 |
| 3 | 10,239 | 0.55 ± 0.05 |  | 0.23 (-0.01, 0.48) | 0.064 |
| 2 | 4,728 | 0.70 ± 0.08 |  | **0.29 (0.02, 0.56)** | **0.038** |
| 0-1 | 866 | 0.96 ± 0.19 |  | **0.50 (0.07, 0.93)** | **0.022** |
| Sleep pattern |  |  |  |  |  |
| Healthy | 11,197 | 0.25 ± 0.05 |  | Reference | - |
| Intermediate | 14,967 | 0.60 ± 0.04 |  | **0.25 (0.11, 0.38)** | **<0.001** |
| Poor | 866 | 0.96 ± 0.19 |  | **0.50 (0.12, 0.88)** | **0.011** |

Models were adjusted for age, sex, education, race, socioeconomic status, healthy lifestyle (smoking status, alcohol intake, physical activity, and social contact), and cardiometabolic disease.

**Table S11—**Associations between healthy sleep pattern and brain age gap (BAG): excluding 2,877 participants from the training set.

|  | No. of participants | LS Mean ± SE |  | BAG |  |
| --- | --- | --- | --- | --- | --- |
|  |  |  |  | β (95% CI) | *P*-value |
| Healthy sleep score  (per 1-unit decrease) | 24,623 | 0.53 ± 0.04 |  | **0.12 (0.05, 0.20)** | **0.001** |
| 5 | 1,986 | 0.29 ± 0.12 |  | Reference | - |
| 4 | 7,944 | 0.31 ± 0.06 |  | -0.05 (-0.32, 0.22) | 0.705 |
| 3 | 9,388 | 0.61 ± 0.06 |  | 0.18 (-0.09, 0.44) | 0.193 |
| 2 | 4,463 | 0.76 ± 0.08 |  | 0.22 (-0.07, 0.52) | 0.1127 |
| 0-1 | 842 | 1.02 ± 0.19 |  | **0.43 (0.01, 0.89)** | **0.045** |
| Sleep pattern |  |  |  |  |  |
| Healthy | 9,930 | 0.31 ± 0.06 |  | Reference | - |
| Intermediate | 13,851 | 0.66 ± 0.05 |  | **0.24 (0.09, 0.38)** | **0.001** |
| Poor | 842 | 1.02 ± 0.19 |  | **0.48 (0.08, 0.87)** | **0.019** |

Models were adjusted for age, sex, education, race, socioeconomic status, healthy lifestyle (smoking status, alcohol intake, physical activity, and social contact), and cardiometabolic disease.

As outlined in “APPENDIX I: Machine learning-based calculation of brain age,” the training set for the brain age estimation model included 3,484 healthy participants, 2,877 of whom remained in the analytical sample for the present study after exclusions for missing sleep data.

**Table S12—**Associations between healthy sleep pattern and brain age gap (BAG): further adjusted for brain MRI assessment centre.

|  | No. of participants | LS Mean ± SE |  | BAG |  |
| --- | --- | --- | --- | --- | --- |
|  |  |  |  | β (95% CI) | *P*-value |
| Healthy sleep score  (per 1-unit decrease) | 27,500 | 0.49 ± 0.03 |  | **0.14 (0.07, 0.21)** | **<0.001** |
| 5 | 2,313 | 0.21 ± 0.11 |  | Reference | - |
| 4 | 9,006 | 0.27 ± 0.06 |  | 0.01 (-0.24, 0.26) | 0.930 |
| 3 | 10,413 | 0.57 ± 0.05 |  | 0.24 (-0.01, 0.49) | 0.054 |
| 2 | 4,870 | 0.73 ± 0.08 |  | **0.31 (0.04, 0.58)** | **0.025** |
| 0-1 | 898 | 0.99 ± 0.18 |  | **0.52 (0.10, 0.95)** | **0.016** |
| Sleep pattern |  |  |  |  |  |
| Healthy | 11,319 | 0.26 ± 0.05 |  | Reference | - |
| Intermediate | 15,283 | 0.62 ± 0.04 |  | **0.25 (0.12, 0.39)** | **<0.001** |
| Poor | 898 | 0.99 ± 0.18 |  | **0.51 (0.13, 0.89)** | **0.008** |

Models were adjusted for age, sex, education, race, socioeconomic status, healthy lifestyle (smoking status, alcohol intake, physical activity, and social contact), cardiometabolic disease, and brain MRI assessment centre.

**Table S13—**Associations between healthy sleep pattern and brain age gap (BAG): further adjusted for body mass index.

|  | No. of participants | LS Mean ± SE |  | BAG |  |
| --- | --- | --- | --- | --- | --- |
|  |  |  |  | β (95% CI) | *P*-value |
| Healthy sleep score  (per 1-unit decrease) | 27,500 | 0.48 ± 0.03 |  | **0.011 (0.04, 0.18)** | **0.001** |
| 5 | 2,313 | 0.21 ± 0.11 |  | Reference | - |
| 4 | 9,006 | 0.27 ± 0.06 |  | -0.00 (-0.24, 0.25) | 0.982 |
| 3 | 10,413 | 0.57 ± 0.05 |  | 0.21 (-0.04, 0.45) | 0.097 |
| 2 | 4,870 | 0.73 ± 0.08 |  | **0.24 (-0.02, 0.51)** | **0.079** |
| 0-1 | 898 | 0.99 ± 0.18 |  | **0.45 (0.02, 0.88)** | **0.038** |
| Sleep pattern |  |  |  |  |  |
| Healthy | 11,319 | 0.26 ± 0.05 |  | Reference | - |
| Intermediate | 15,283 | 0.62 ± 0.04 |  | **0.22 (0.09, 0.35)** | **0.001** |
| Poor | 898 | 0.99 ± 0.18 |  | **0.45 (0.08, 0.83)** | **0.019** |

Models were adjusted for age, sex, education, race, socioeconomic status, healthy lifestyle (smoking status, alcohol intake, physical activity, and social contact), cardiometabolic disease, and body mass index.

**Table S14—**Associations between healthy sleep pattern and brain age gap (BAG): further adjusted for APOE ɛ4.

|  | No. of participants | LS Mean ± SE |  | BAG |  |
| --- | --- | --- | --- | --- | --- |
|  |  |  |  | β (95% CI) | *P*-value |
| Healthy sleep score  (per 1-unit decrease) | 27,500 | 0.49 ± 0.03 |  | **0.14 (0.06, 0.21)** | **<0.001** |
| 5 | 2,313 | 0.21 ± 0.11 |  | Reference | - |
| 4 | 9,006 | 0.27 ± 0.06 |  | -0.02 (-0.29, 0.25) | 0.875 |
| 3 | 10,413 | 0.57 ± 0.05 |  | 0.20 (-0.07, 0.47) | 0.141 |
| 2 | 4,870 | 0.73 ± 0.08 |  | **0.31 (0.01, 0.60)** | **0.043** |
| 0-1 | 898 | 0.99 ± 0.18 |  | **0.44 (0.01, 0.91)** | **0.040** |
| Sleep pattern |  |  |  |  |  |
| Healthy | 11,319 | 0.26 ± 0.05 |  | Reference | - |
| Intermediate | 15,283 | 0.62 ± 0.04 |  | **0.25 (0.11, 0.40)** | **0.001** |
| Poor | 898 | 0.99 ± 0.18 |  | **0.46 (0.05, 0.87)** | **0.027** |

Models were adjusted for age, sex, education, race, socioeconomic status, healthy lifestyle (smoking status, alcohol intake, physical activity, and social contact), cardiometabolic disease, and APOE ɛ4 carrier status.
